# PAR-1 is a novel mechano-sensor transducing laminar flow-mediated endothelial signaling

**DOI:** 10.1038/s41598-018-33222-3

**Published:** 2018-10-11

**Authors:** Suji Kim, Jung-Hwa Han, Dae-Hwan Nam, Geun-Young Kim, Jae Hyang Lim, Jae-Ryong Kim, Chang-Hoon Woo

**Affiliations:** 1 Department of Pharmacology, Yeungnam University College of Medicine, 317-1 Daemyung-dong, Daegu, Republic of Korea; 2 Smart-Ageing Convergence Research Center, Yeungnam University College of Medicine, 317-1 Daemyung-dong, Daegu, Republic of Korea; 3 Predictive Model Research Center, Korea Institute of Toxicology, Korea Research Institute of Chemical Technology, Daejeon, Republic of Korea; 4 Jeju National Quarantine Station, Centers for Disease Control & Prevention, Jeju, Republic of Korea; 5 Department of Microbiology, Ewha Womans University School of Medicine, 911-1 Mok-dong, Seoul, Republic of Korea

## Abstract

Recent studies have indicated that protease-activated receptor-1 (PAR-1) is involved in cytoprotective and anti-inflammatory responses in endothelial cells (ECs). However, the role of PAR-1 in laminar flow-mediated atheroprotective responses remains unknown. Herein, we investigated whether PAR-1 regulates laminar flow-mediated mechanotransduction in ECs. Confocal analysis showed that PAR-1 was internalized into early endosomes in response to laminar flow. In addition, flow cytometry analysis showed that cell surface expression of PAR-1 was reduced by laminar flow, suggesting that PAR-1 was activated in response to laminar flow. Depletion of PAR-1 using human PAR-1 siRNA inhibited unidirectional laminar flow-mediated actin stress fiber formation and cellular alignment as well as atheroprotective gene expressions in HUVECs. Moreover, PAR-1 knockdown inhibited laminar flow-stimulated eNOS phosphorylation, and inhibited the phosphorylations of Src, AMPK, ERK5 and HDAC5. Furthermore, PAR-1 depletion inhibited laminar flow-mediated anti-inflammatory responses as demonstrated by reduced TNFα-induced VCAM-1 expression and by monocyte adhesion to HUVECs, and prevented laminar flow-mediated anti-apoptotic response. An investigation of the role of PAR-1 in vasomotor modulation using mouse aortic rings revealed that acetylcholine-induced vasorelaxation was diminished in PAR-1 deficient mice compared to littermate controls. Taken together, these findings suggest that PAR-1 be viewed as a novel pharmacologic target for the treatment of vascular diseases, including atherosclerosis.

## Introduction

Atherosclerosis preferentially involves regions prone to low and/or disturbed blood flow at vessel branch points and the outer walls of bifurcations, and conversely regions exposed to steady, high levels of laminar blood flow are relatively protected from atherosclerosis^1–4^. When endothelial cells (ECs) are subjected to laminar flow, many of their functions including vascular remodeling, coagulation, proliferation, apoptosis, migration, and permeability are affected^5,6^. ECs transduce responses to laminar flow through mechano-sensors, which include, integrins, ion channels, vascular endothelial growth factor receptor 2 (VEGFR-2), lipid rafts, G protein coupled receptors (GPCRs), and G protein**s**^7–10^. For instance, laminar flow activates α_v_β_3_ integrins and the VEGF receptor Flk-1^11,12^. G proteins have been shown to participate in laminar flow-dependent endothelial signaling and NO production^13^, and it has been reported that pertussis toxin (PTX; inhibitor of the α_i_ subunit of heterotrimeric G protein) down-regulates laminar flow-induced MAP kinase activation in ECs^14^. In addition, G_q_ and G_11_ have recently been shown to mediate laminar flow-induced endothelial responses, including NO formation and the regulations of vascular tone and blood pressure^15^. However, the molecular mechanism linking GPCRs and laminar flow signaling to nuclei has not been previously addressed.

Laminar flow activates extracellular signal-regulated kinase 5 (ERK5) that promotes the atheroprotective signaling pathway^16^. Laminar flow-induced ERK5 activation mediates Krüppel-like factor 2 (KLF2) that induces endothelial nitric oxide synthase (eNOS) expression and mediates anti-inflammatory responses^17,18^. Also, laminar flow activates NF-E2 related factor 2 (Nrf2), which contributes to atheroprotective effects in an ERK5 dependent manner^19^. On the other hand, in a recently study, it was shown laminar flow-mediated signalings are negatively regulated by histone deacetylase 5^20^ (HDAC5; a member of the class IIa histone deacetylases and negative regulator of myocyte enhancer factor-2 (MEF2), a downstream target of the laminar flow-induced ERK5 activating pathway)^21^. Having been is phosphorylated by various kinases, nuclear HDAC5 translocates to cytosol^21,22^. Consequently, laminar flow-induced phosphorylation and nuclear export of HDAC5 increases MEF2 transcriptional activity, which leads to the expressions of KLF2 and eNOS and prevents inflammatory responses^20^.

PAR-1 is responsive to activating protease and is activated via an irreversible proteolytic mechanism^23–25^. Proteases bind to and cleave the N-terminal exodomain of PAR-1 to expose a tethered ligand, which subsequently binds to cleaved PAR-1 and activates the receptor signaling cascade^26^. In particular, after occupancy of endothelial protein C receptor (EPCR) by protein C in ECs the PAR-1 signaling pathway elicits anti-coagulant, anti-inflammatory, and cytoprotective responses^27^. During development, PAR-1 contributes to embryonic blood vessel development by regulating endothelial cell function^28^. In addition, PAR-1 modulates vasomotor activity in various blood vessels. In isolated blood vessels, activation of endothelial PAR-1 triggers vasorelaxation via NO production^29–31^. Interestingly, one study demonstrated that laminar flow reduced the cell surface expression of PAR-1^32^, but the mechanism whereby PAR-1 acts as a mechano-sensor in the laminar flow-mediated atheroprotective signaling pathway has not been studied. Accordingly, in the present study, we investigated whether PAR-1 acts as a mechano-sensor in the laminar flow-mediated atheroprotective signaling pathway.

In this study, we found that PAR-1 is activated by laminar flow and regulates laminar flow-induced atheroprotective gene expression dependent on Src, AMPK, ERK5, HDAC5 and eNOS in EC. In addition, activated PAR-1 was shown to promote anti-inflammatory and anti-apoptotic responses mediated by laminar flow. We also demonstrated that acetylcholine-induced vasorelaxation was diminished in aortic rings of PAR-1 KO compared to littermate controls. These data provide evidence that PAR-1 is a mechano-sensor for laminar flow which mediates anti-atherosclerotic responses in EC.

## Results

### Exposure to laminar flow led to PAR-1 internalization

**Figure 1:**
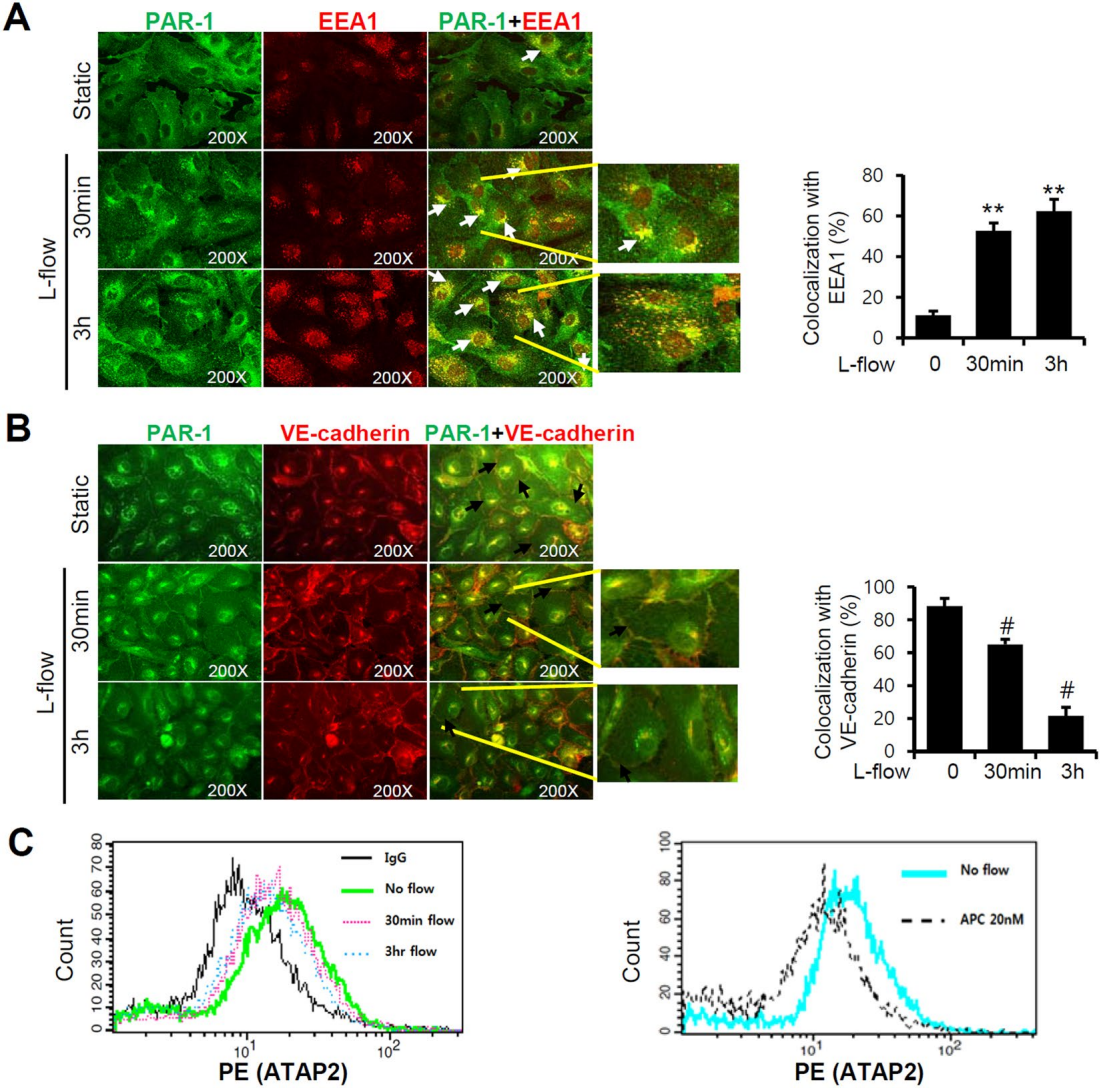
Laminar flow induces PAR-1 internalization in HUVECs. To visualize PAR-1 internalization by HUVECs, cells were exposed to laminar flow (12 dyne/cm^2^) for 30 minutes or 3 hours and subjected to immunostaining. (**A**,**B**) PAR-1 specific staining (left panel). EEA-1 (middle panel of A) or VE-cadherin staining (middle panel of B). Right panels present merged images. White arrow indicates association between PAR-1 and EEA1. (**A**) For PAR-1, localization of the receptors in the untreated cells was dominant at the cell membrane (black arrows of **B**). In the static condition, PAR-1 is located at the cell membrane and colocalized with VE-cadherin (white arrows in merged images). Images were observed under a fluorescence microscope at an original magnification of ×200. The graph shows percentage of PAR-1 immunoreactivity that was colocalized with EEA1 or VE-cadherin. Results are presented as the means ± SDs (*n* = 3). ^**^P < 0.01; ^#^P < 0.05; ^##^P < 0.01; versus with static controls. (**C**) PAR-1 protein levels on cell surfaces were assessed by flow cytometry. After exposure to laminar flow for 30 minutes or 3 hours, HUVECs were stained with monoclonal anti-PAR-1 antibody (ATAP2) or mouse IgG antibody as a negative control. The positive control was treated with 20 nM APC for 1 hour. Results are representative of three independent experiments. Cells were used for experiments at passage 4, 5 and 6.

Previous reports have shown PAR-1 is activated by serine proteases such as thrombin, plasmin, factor Xa, and activated protein C (APC)^33^. Activated PAR-1 then rapidly internalizes from cell surface and translocates to early endosome. Thus, we examined whether exposure to laminar flow stimulates PAR-1 internalization by exposing HUVECs to laminar flow for different times. In non-treated control cells, PAR-1 was primarily found on cell surface colocalized with VE-cadherin, a plasma membrane marker of endothelial cells (Fig. 1B). However, after cells had been exposed to laminar flow, PAR-1 was internalized in a time-dependent manner and translocated to cytosol, where it colocalized with EEA1, an early endosomal marker (Fig. 1A). Next, we examined affinity of anti-PAR-1 antibody (ATAP2) binding to HUVEC surfaces by flow cytometer. After cell had been exposed to laminar flow, ATAP2 binding affinity to surface PAR-1 was found to be significantly down-regulated in a time-dependent manner like as activated protein C (APC) which is an endogenous agonist of PAR-1 (Fig. 1C). These observations suggest that laminar flow activates PAR-1.

### PAR-1 is required for alignment in laminar flow

**Figure 2:**
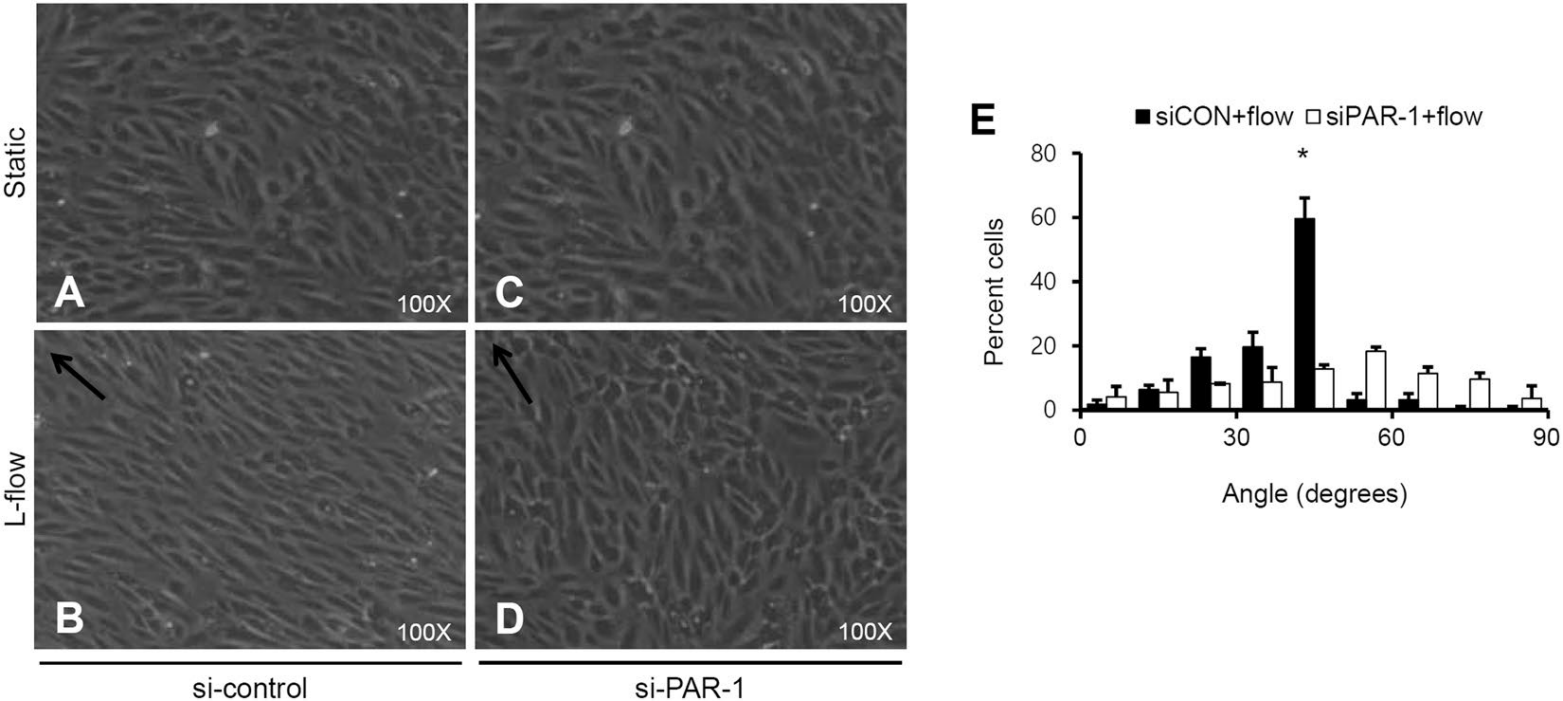
Effect of PAR-1 siRNA on laminar flow-induced alignment in HUVECs. HUVECs were transfected with 100 pmol/L of control siRNA or PAR-1 siRNA for 2 days. (**A**,**C**) Monolayer in static condition. (**B**,**D**) Confluent endothelial cells after 24 hours of exposure to laminar flow at 12 dyne/cm^2^. Morphology changes were measured by microscope at an original magnification of ×100. Arrow indicates direction of laminar flow. Images are representative of three independent experiments. (**E**) The bar graph showed the degree of cell orientation under laminar flow. The maximal percentage of cells aligned at around 30° to 50° angle (direction of flow) under laminar flow in EC. The asterisk represents statistical significance of percent cells aligned in the direction of laminar flow (^*^P < 0.05). Cells were used for experiments at passage 4, 5 and 6.

Laminar flow has been linked to alignment of EC in the direction of flow^34,35^. To study whether PAR-1 regulates flow-induced EC alignment, confluent cultured endothelial cells were transfected with control RNA or PAR-1 siRNA for 2 days and then exposed to 12 dynes/cm^2^ of laminar flow for 24 hours. The shape of endothelial cells was changed by exposure to unidirectional flow of 12 dynes/cm^2^ in laminar flow for 24 hours (Fig. 2B,E). In contrast, PAR-1 knockdown using PAR-1 specific siRNA markedly dysregulates alignment of EC in response to the laminar flow (Fig. 2D,E). These results indicate that PAR-1 regulates unidirectional flow-induced cell alignment in ECs.

### PAR-1 mediates actin stress fiber (ASF) formation in HUVECs

**Figure 3:**
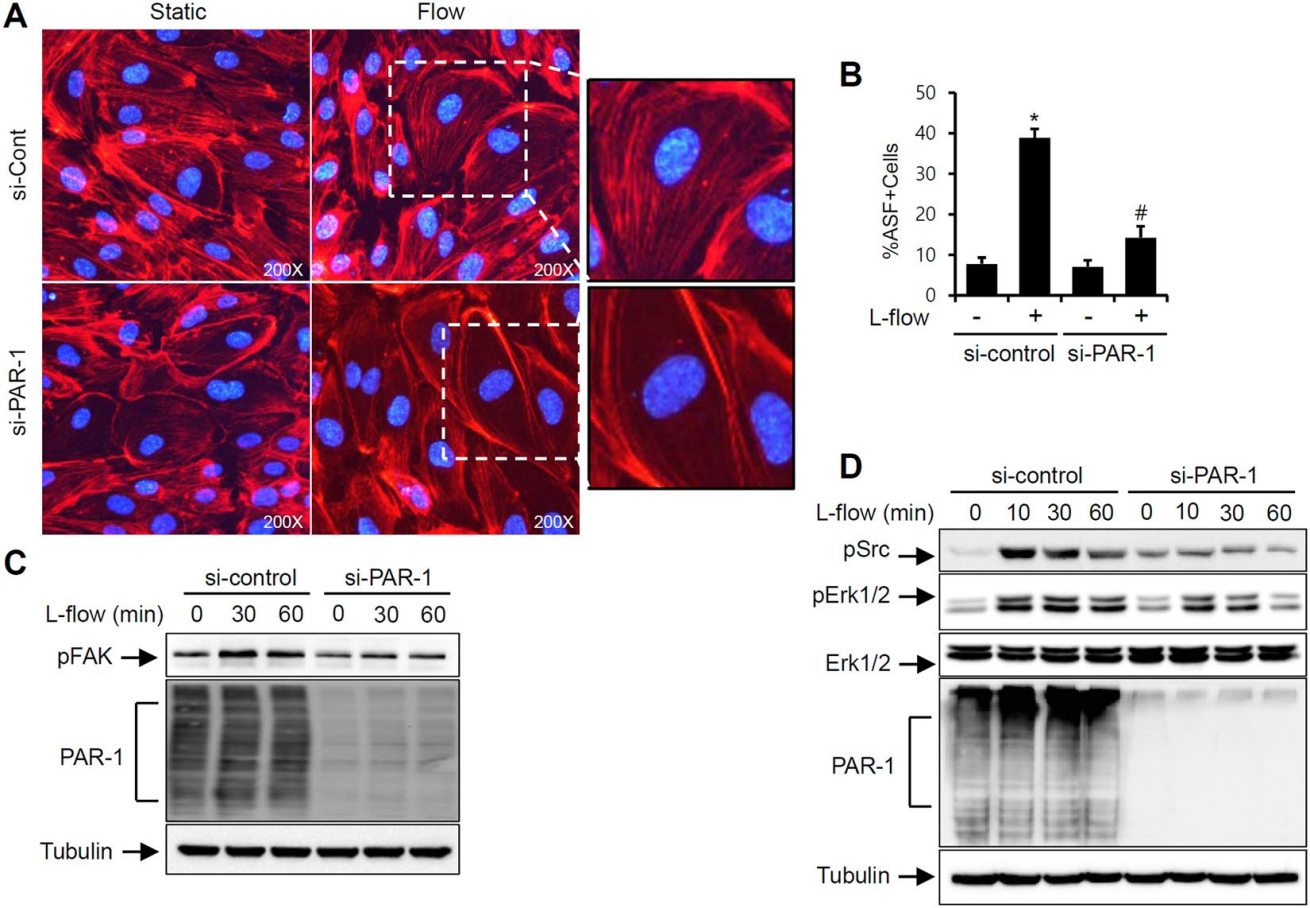
PAR-1 knockdown attenuates laminar flow-induced actin stress fiber formation in ECs. (**A**) HUVECs transfected with control RNA or PAR-1 siRNA were exposed to laminar flow. Representative photomicrographs showing F-actin with Texas Red-X Phalloidin and nucleus with DAPI (original magnification ×200). (**B**) Quantification of actin stress fiber (ASF) is presented by bar graph. (**C**) Cells transfected with control RNA or PAR-1 siRNA (100 pmol/L) for 2 days were exposed to laminar flow for 30 or 60 minutes. Protein levels of pFAK, PAR-1, and tubulin were determined by Western blotting against specific antibodies. (**D**) Cells transfected with control or PAR-1 siRNA (100 pmol/L) for 2 days were exposed to laminar flow for 10, 30 and 60 minutes. Protein levels of pSrc, pErk1/2, Erk1/2, PAR-1, and tubulin were assayed by Western blotting against specific antibodies. The results shown are representative of three independent experiments. Cells were used for experiments at passage 4, 5 and 6.

Laminar flow induces actin stress fiber (ASF) formation inducing the realignment of focal adhesions which is related to spatial and temporal responses of the associated protein including focal adhesion kinase (FAK)^36^. To investigate if PAR-1 regulates laminar flow-induced ASF formation, confluent cultured endothelial cells were transfected with control RNA or PAR-1 siRNA for 2 days and then exposed to 12 dynes/cm^2^ of laminar flow followed by phalloidin staining against F-actin. Laminar flow significantly increased the ASF formation (Fig. 3A,B). In contrast, PAR-1 knockdown using PAR-1 specific siRNA markedly dysregulates ASF formation of EC in response to the laminar flow (Fig. 3A,B). These results suggest that PAR-1 regulates laminar flow-mediated integrin-dependent signaling and ASF formation.

Laminar shear stress is known to induce phosphorylation of FAK, a major focal contact protein, which is regulated by integrin engagement^37,38^. A recent study showed that P2Y2 receptor acts as a mechano-sensor and modulates laminar shear stress-induced ASF formation in endothelial cells^36^. To investigate whether PAR-1 regulates the laminar flow-induced activation of FAK, HUVECs transfected with siRNA against PAR-1 were exposed to flow for 30 or 60 minutes. As shown in new Fig. 3C, laminar flow-induced FAK phosphorylation was markedly diminished in endothelial cells transfected with siRNA against PAR-1, suggesting the role of PAR-1 in laminar flow signal to cytoskeletal rearrangement.

It has been known that laminar shear stress transduce mechanotransdution through receptor kinase and subsequent Src kinase activation^5,39^. In particular, laminar flow increases phosphorylation of Src at early time points^40^. To address the role of PAR-1 in laminar flow-induced Src activation, we determined the Src phosphorylation in response to laminar flow at the various time points. As shown in Fig. 3D, laminar flow-induced Src phosphorylation was peaked at 10 mins and markedly inhibited in endothelial cells transfected with siRNA against PAR-1 compared to control siRNA. In addition, we found similar response with ERK1/2 phosphorylation which is a downstream effector of Src (Fig. 3D). This result suggests that PAR-1 acts as a mechano-sensor for laminar flow and modulates Src-dependent signaling pathway in endothelial cells.

### PAR-1 is responsible for laminar flow-induced KLF2- and Nrf2-dependent gene expressions

**Figure 4:**
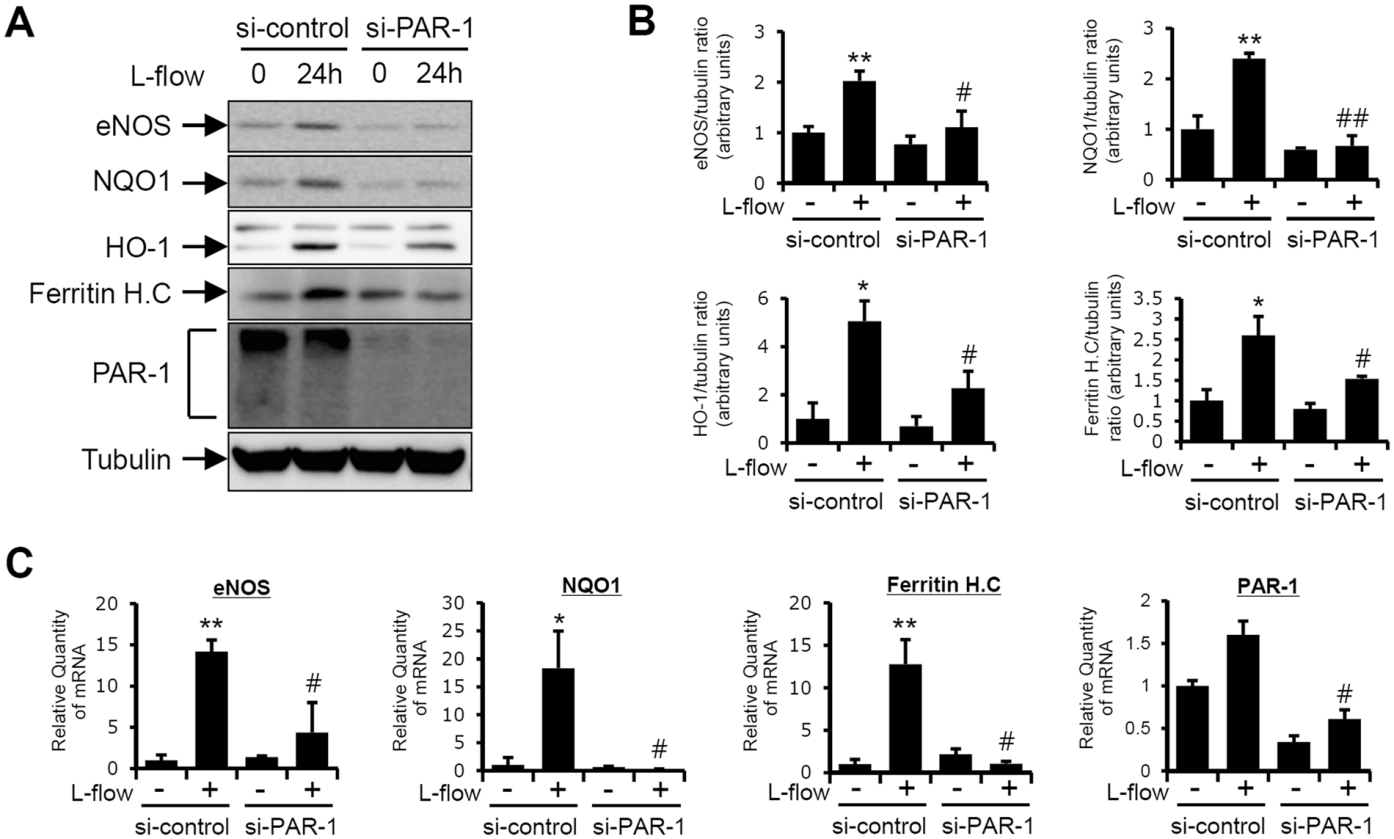
The involvement of PAR-1 in laminar flow-induced expressions of KLF2- and Nrf2-dependent genes. HUVECs were transfected with 100 pmol/L of control siRNA or PAR-1 siRNA for 2 days and then exposed to laminar flow for 24 hours. (**A**) Protein levels were determined by immunoblotting with specific antibodies against eNOS, NQO1, HO-1, PAR-1, and anti-tubulin. (**B–F**) mRNA levels of eNOS, NQO1, ferritin heavy chain (*Ferritin* H.C) and PAR-1 was determined by RT-qPCR. Relative expression levels were normalized versus GAPDH. Results are representative of three independent experiments that yielded similar results. Results are presented as the means ± SDs (*n* = 3). ^*^P < 0.05; ^**^P < 0.01 compared with control siRNA transfected HUVECs. ^#^P < 0.05; ^##^P < 0.01 compared with control siRNA transfected HUVECs under laminar flow conditions (12 dynes/cm^2^). Cells were used for experiments at passage 4, 5 and 6.

To determine the effect of PAR-1 on laminar flow-mediated endothelial signaling, we first investigated whether it affects laminar flow-inductions of the KLF2 and Nrf2 downstream genes. HUVECs were transfected with control or PAR-1 siRNA for 2 days and then exposed to 12 dynes/cm^2^ of laminar flow for 24 hours. Laminar flow was found to enhance protein expression of eNOS which is a downstream target of KLF2. In addition, laminar flow induced protein expressions of Nrf2-dependent genes such as NQO1, HO-1, and Ferritin heavy chain in control siRNA-transfected cells. In contrast, PAR-1 knockdown using PAR-1 specific siRNA markedly inhibited the laminar flow-induced protein expressions of KLF2- and Nrf2-dependent genes (Fig. 4A,B). Interestingly, PAR-1 was observed as a broad migration band with a molecular mass in control siRNA-transfected cells. It has been reported that this broad migration band is due to N-linked glycosylation^41^. After treatment with siRNA the protein expression of PAR-1 was markedly reduced, and the flow-induced mRNA expressions of KLF2- and Nrf2-targeted genes were significantly inhibited (Fig. 4C). These results suggest that PAR-1 contributes to the laminar flow-induced KLF2- and Nrf2-dependent signaling pathways.

### PTX inhibited the flow-induced phosphorylations of Akt, AMPK, ERK5 and HDAC5

**Figure 5:**
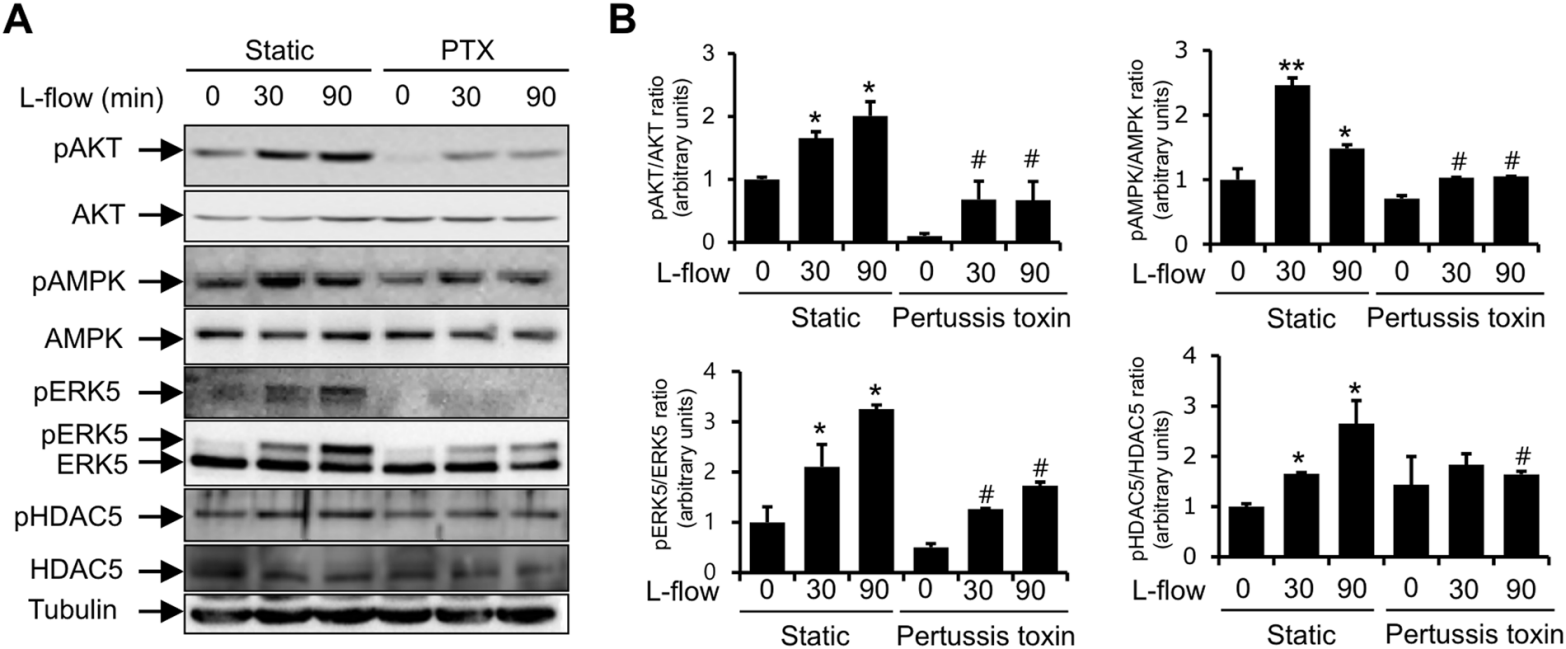
Pertussis toxin inhibits the laminar flow-induced phosphorylations of Akt, AMPK, ERK5, and HDAC5 in HUVECs. Cells were preincubated with 100 ng/ml of pertussis toxin for 1 hour and then exposed to laminar flow (12 dynes/cm^2^) for 30 or 90 minutes. (**A**) Protein levels were determined by immunoblotting using specific antibodies against pAKT, AKT, pAMPK, AMPK, pERK5, ERK5, pHDAC5, HDAC5, and anti-tubulin. Results are representative of three independent experiments that yielded similar results, and are presented as means ± SDs (*n* = 3). ^*^P < 0.05; ^**^P < 0.01; versus with static controls. ^#^P < 0.05 vs. HUVECs exposed to laminar flow (12 dynes/cm^2^). Cells were used for experiments at passage 4, 5 and 6.

It has been established that heterotrimeric G proteins and GPCRs play roles in the sensing of the laminar flow^7^, and that thrombin-induced PAR-1 activation is regulated by G_q_ or G_12/13_, whereas the ligand occupancy of EPCR couples PAR-1 to Gα_*i*_ protein and leads to cytoprotective responses in ECs^42,43^. To determine the role of heterotrimeric G proteins in laminar flow-mediated endothelial signaling, we investigated whether G proteins affect the laminar flow-stimulated activation of kinases in HUVECs. Pharmacological inhibition of the α_i_ subunit of G proteins using pertussis toxin (PTX) markedly inhibited the laminar flow-induced activations of Akt, AMPK and ERK5 (Fig. 5A,B).

Recently it was reported that laminar flow-stimulated HDAC5 phosphorylation mediates the expressions of KLF2 and eNOS, and modulates laminar flow-induced anti-inflammatory responses^20^, and that HDAC5 negatively regulates the signaling pathway of myocyte enhancer factor-2 (MEF-2), which is deacetylated by HDAC5^21^. Thus, it appears that these signaling pathways play an important role in negative regulation of laminar flow signaling. For this reason, we investigated if G protein signaling pathway involvement in laminar flow signaling occurs through HDAC5 signaling rather than kinase signaling pathways. To examine the molecular whereby G proteins mediate laminar flow-induced HDAC5 phosphorylation, Gα_i_ protein inhibitor PTX was administered to HUVECs during exposure to laminar flow. As shown in Fig. 5A,B, the laminar flow-induced phosphorylation of HDAC5 was greatly abolished by PTX. Taken together, these results suggest that Gα_i_ protein mediates PAR-1-mediated laminar flow by signaling to Akt, AMPK, ERK5, and HDAC5 in ECs.

### PAR-1 mediated the laminar flow-induced phosphorylations of AMPK, ERK5, HDAC5, and eNOS

**Figure 6:**
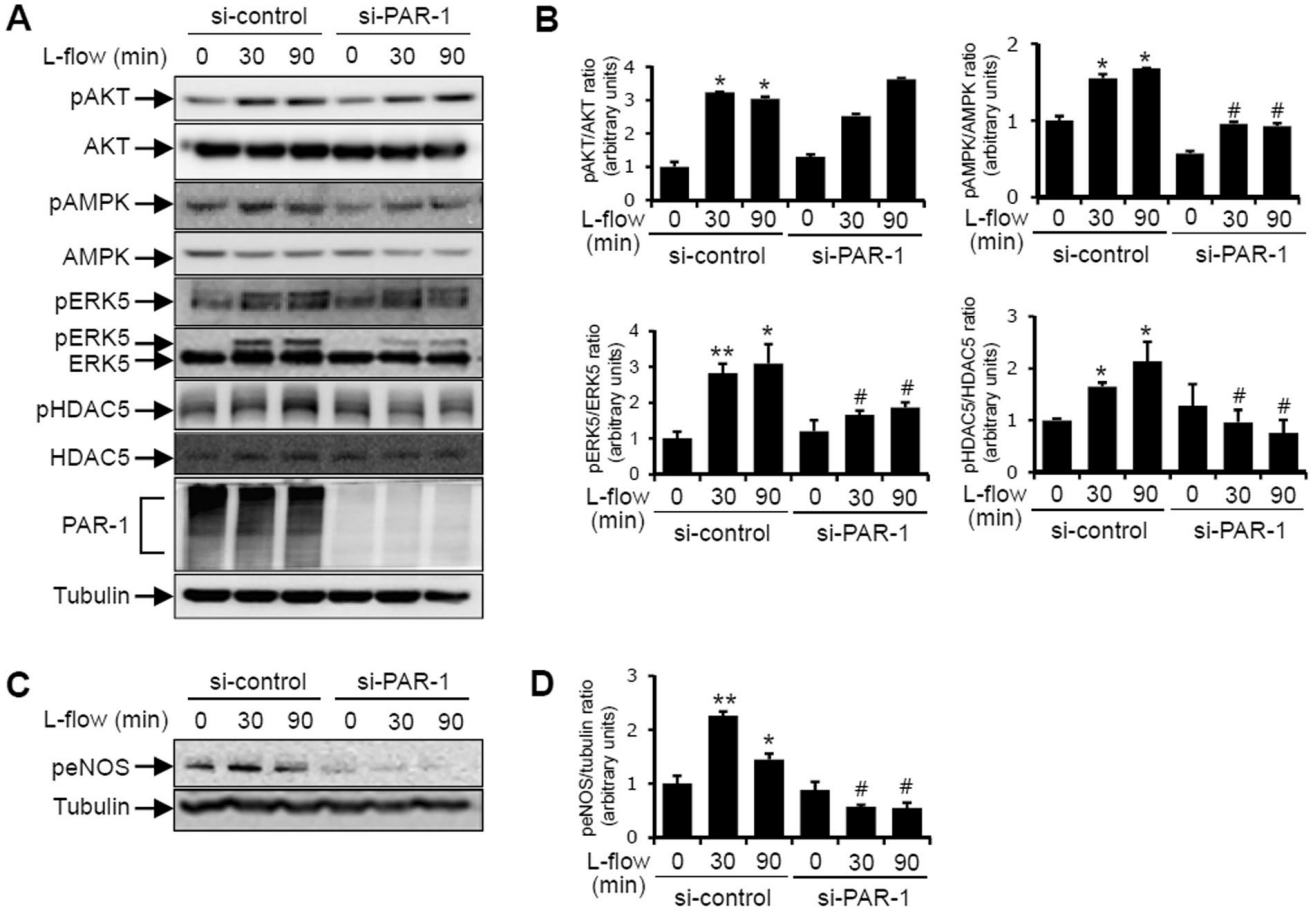
The effects of PAR-1 siRNA on the laminar flow-induced phosphorylations of Akt, AMPK, ERK5, HDAC5, and eNOS in HUVECs. Cells transfected with control or PAR-1 siRNA (100 pmol/L) for 2 days were exposed to laminar flow for 30 or 90 minutes. (**A**, **B**) Protein levels of pAKT, AKT, pAMPK, AMPK, pERK5, ERK5, pHDAC5, HDAC5, PAR-1, and tubulin were assayed by Western blotting. (**C**, **D**) Protein levels of peNOS and tubulin were determined by immunoblotting with specific antibodies, respectively. The results shown are representative of three independent experiments. ^*^P < 0.05; ^**^P < 0.01 vs. control siRNA transfected HUVECs. ^#^P < 0.05 vs. control siRNA transfected HUVECs exposed to laminar flow (12 dynes/cm^2^). Cells were used for experiments at passage 4, 5 and 6.

We next sought to identify the mechanisms by which PAR-1 mediates the laminar flow-dependent activation of kinases. It has been well established that laminar flow activates various kinases, including Akt, AMPK, ERK5, and HDAC5^5,44^. To determine whether PAR-1 regulates the laminar flow-induced phosphorylations of Akt, AMPK, ERK5, and HDAC5, HUVECs transfected with siRNA against PAR-1 were exposed to flow for 30 or 90 minutes. As shown in Fig. 6A,B, the phosphorylations of AMPK, ERK5 and HDAC5 were reduced under these flow conditions in PAR-1 siRNA transfected cells. In addition, ERK5 activation was detected by a band shift which is a characteristic of phosphorylated ERK5. However, flow-induced Akt phosphorylation was not affected by PAR-1 knockdown using PAR-1-specific siRNA. These findings show that PAR-1 promotes the flow-mediated activations of AMPK, ERK5 and HDAC5 signaling pathways.

The nitric oxide (NO) produced by eNOS plays a critical role in vascular function including vascular relaxation. Following exposure to laminar flow, eNOS^Ser 1177^ is phosphorylated and NO produced^45^. We examined the effect of PAR-1 on phosphorylation of eNOS serine 1177 in ECs exposed to laminar flow for 30 or 90 minutes and found that PAR-1 siRNA markedly inhibited laminar flow-induced eNOS^Ser 1177^ phosphorylation (Fig. 6C,D). These results indicate that PAR-1 plays a critical role in the laminar flow-dependent phosphorylations of AMPK, ERK5, HDAC5, and eNOS.

### PAR-1 contributed to laminar flow-mediated anti-inflammatory effects on TNFα-induced inflammation

**Figure 7:**
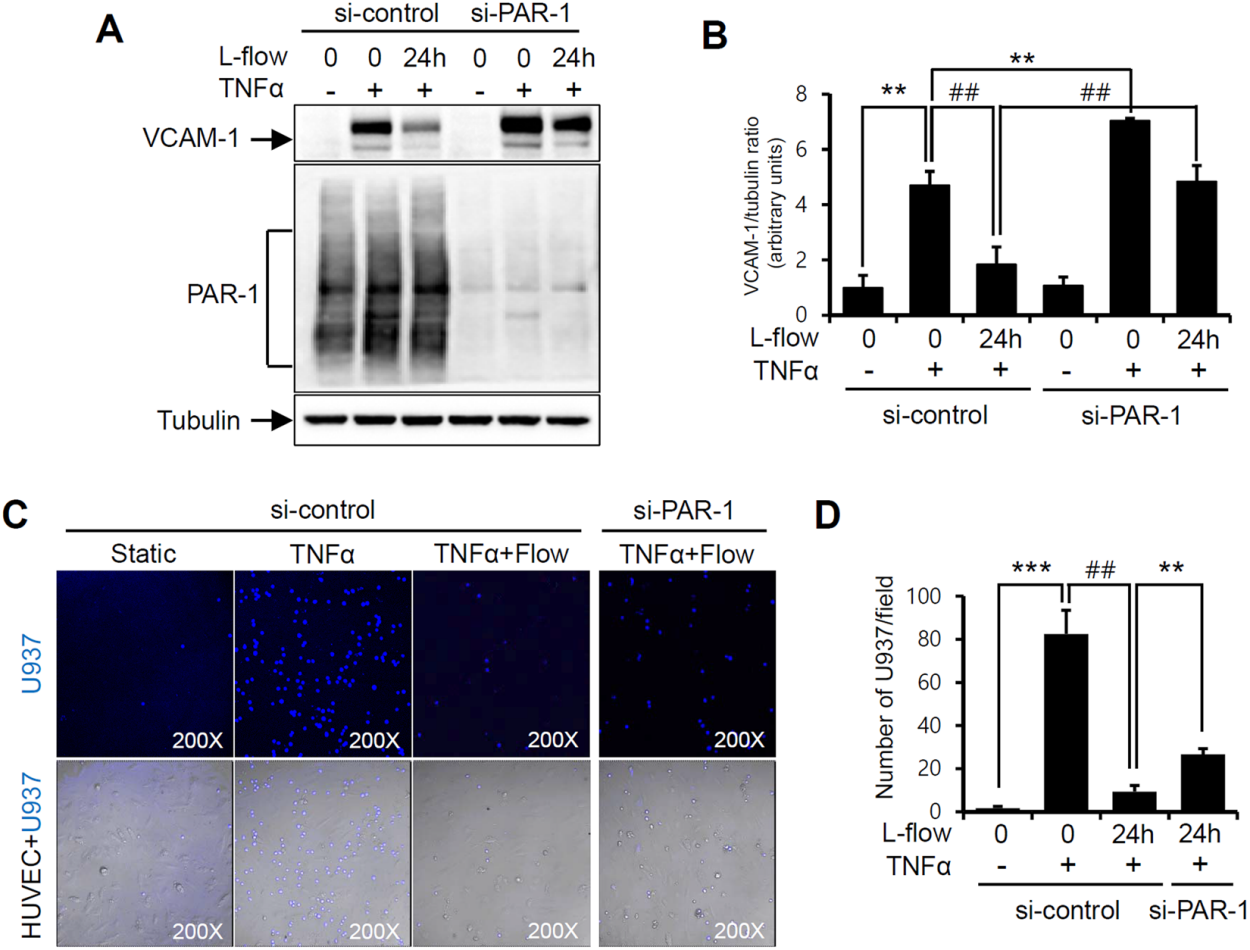
Laminar flow-induced anti-inflammatory responses by PAR-1. HUVECs transfected with 100 pmol/L of control or PAR-1 siRNA were exposed to laminar flow (12 dynes/cm^2^) 24 hours in the presence or absence of TNFα (10 ng/ml). (**A**) Cell lysates were immunoblotted using anti-VCAM-1, anti-PAR-1 (ATAP2), and anti-tubulin antibodies. The asterisk (*) indicates a non-specific band detected by the anti-VCAM-1 antibody. (**B**) Relative protein expressions of VCAM-1 normalized by tubulin are presented in the bar graph. (**C**) U937 cells were stained with 5 μM Vybrant DiD for 20 minutes at 37 °C, and then incubated with HUVECs for 1 hr. The adherent monocytes were visualized at a magnification of ×200 under a confocal fluorescence microscope. (**D**) Adhesion cells were counted and presented in the bar graph. ^*^P < 0.05; ^**^P < 0.01; ^***^P < 0.001 vs. control siRNA transfected HUVECs. ^#^P < 0.05; ^##^P < 0.01 vs. control siRNA transfected HUVECs treated with TNFα. Cells were used for experiments at passage 4, 5 and 6.

PAR-1 is involved in APC-mediated anti-inflammatory responses that reduce the expressions of vascular adhesion molecules^27^. Thus, we sought to determine the role of PAR-1 in laminar flow-mediated anti-inflammatory responses. HUVECs transfected with PAR-1 siRNA for 2 days were treated with the pro-inflammatory cytokine tumor necrosis factor alpha (TNFα) under laminar flow conditions for 24 hours. The expression of vascular cell adhesion molecule 1 (VCAM-1) was observed immediately after TNFα treatment, but was markedly less in control siRNA-transfected HUVECs. However, laminar flow failed to inhibit TNFα-induced VCAM-1 expression in PAR-1 siRNA transfected cells (Fig. 7A,B).

To determine whether PAR-1 regulates TNFα-induced monocyte adhesion to HUVECs, we used a monocyte adhesion assay using the fluorescence labeling technique and U937 monocytes. HUVECs transfected with control siRNA or PAR-1 siRNA under laminar flow conditions for 24 hours were treated with TNFα and incubated with monocytes for 1 hour. Laminar flow significantly reduced monocyte adhesion to TNFα-stimulated HUVECs, and monocyte adhesion was greater for TNFα-stimulated HUVECs transfected with PAR-1 siRNA (Fig. 7C,D). These results suggest PAR-1 activation regulates laminar flow-induced anti-inflammatory responses.

### PAR-1 was involved in laminar flow-mediated anti-apoptotic response in HUVECs

**Figure 8:**
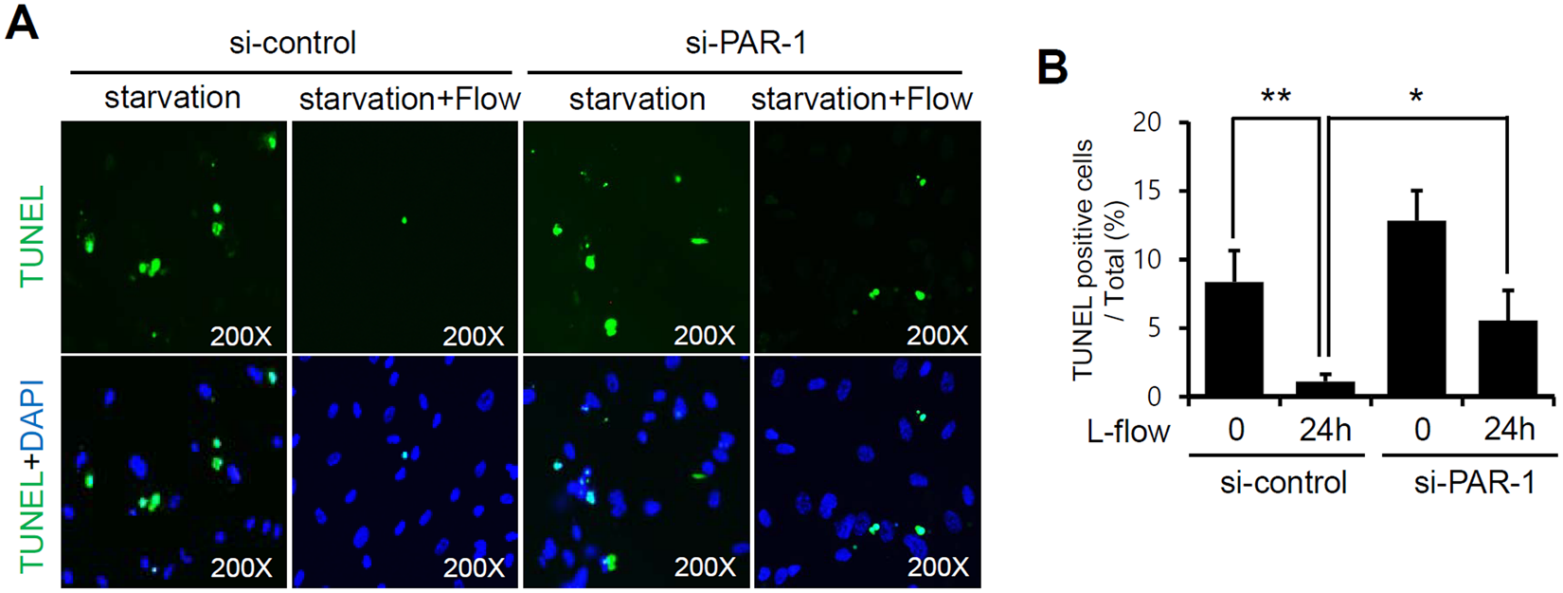
PAR-1 is responsible for laminar flow-induced anti-apoptotic response against serum deprivation. HUVECs transfected with control or PAR-1 siRNA were starved serum and then exposed to laminar flow 24 hours. Apoptotic cells were detected using a TUNEL assay and a fluorescence microscope (×200). Bar graphs present percentage of TUNEL positive cells from total HUVECs counted. Results are presented as the means ± SDs of three independent experiments. ^*^P < 0.05; ^**^P < 0.01 vs. control siRNA transfected HUVECs. ^#^P < 0.05 vs. control siRNA transfected HUVECs under laminar flow (12 dynes/cm^2^). Cells were used for experiments at passage 4, 5 and 6.

To confirm the biological relevance of laminar flow-induced PAR-1 activation, a TUNEL assay was used to assess HUVEC apoptosis. After siRNA transfection for 2 days, HUVECs were serum-starved in order to induce apoptosis and then exposed to laminar flow for 24 hours. As shown in Fig. 8, laminar flow markedly inhibited serum starvation-induced apoptosis, and its inhibitory effect was abolished by PAR-1 knockdown. These results indicate that PAR-1 is required for laminar flow-mediated cytoprotective effects in HUVECs.

### PAR-1 deficiency exacerbated aortic endothelial dysfunction

**Figure 9:**
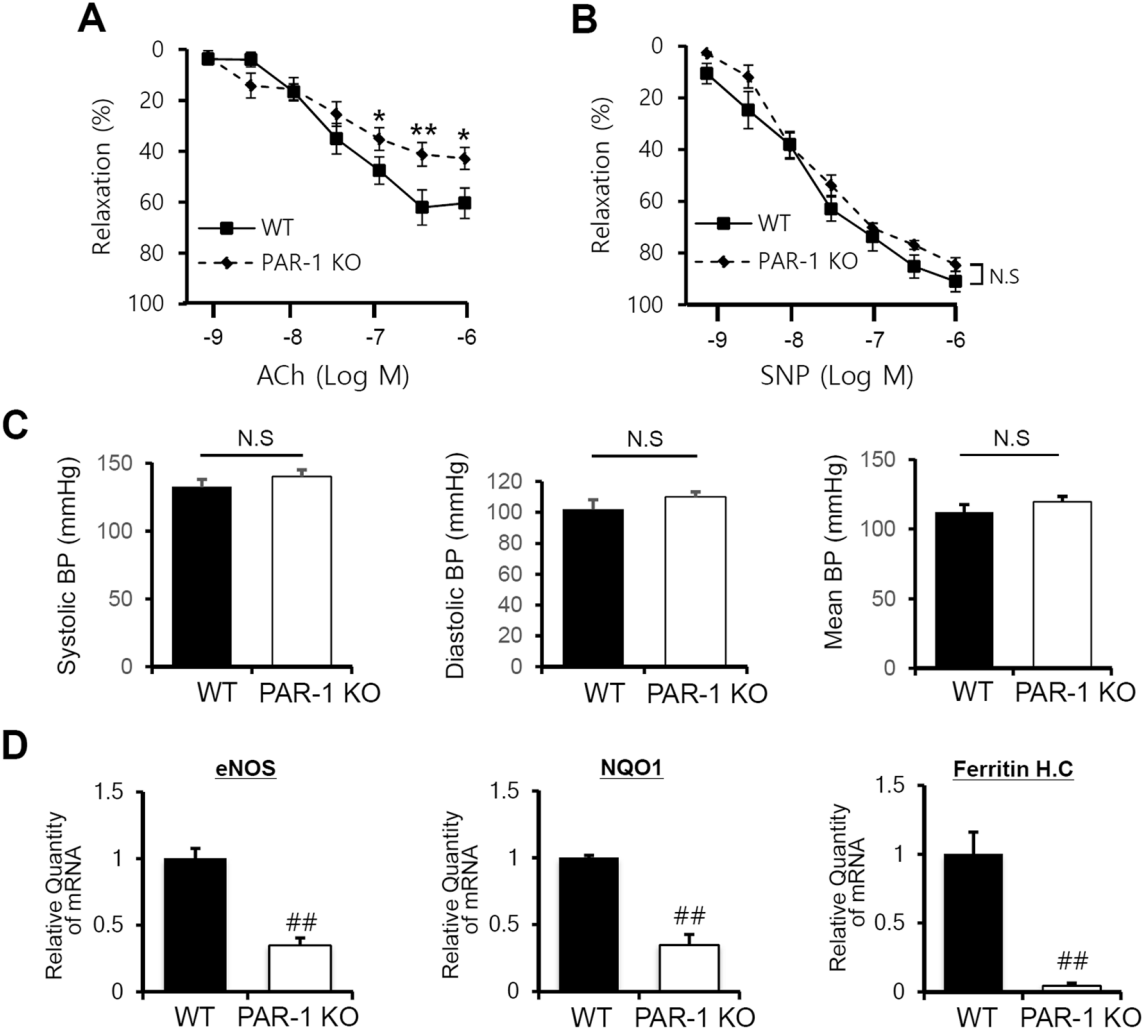
PAR-1 deficiency induces aortic endothelial dysfunction. The effect of acetylcholine (Ach) on U46619-induced contraction in mouse aorta. (**A**) Mouse aorta rings of WT and PAR-1 KO were preincubated with U46619 (10^−8.5^) or Ach (10^−9^ to 10^−6^) to determine the influence of PAR-1 on this response. The results shown are representative of three independent experiments. ^*^P < 0.05; ^**^P < 0.01 vs. WT. (**B**) Dose-responses to sodium nitroprusside (SNP) of U46619 precontracted aortic rings of WT and PAR-1 KO mice. Results are presented as the means ± SDs. n = 7 per group. ^*^P < 0.05; ^**^P < 0.01 versus WT mice. N.S; not significant. (**C**) Basal blood pressure in WT (n = 6) and PAR-1 KO mice (n = 6). Graph shows systolic and diastolic, mean arterial blood pressure. N.S; not significant. (**D**) mRNA levels of eNOS, NQO1 and ferritin heavy chain (*Ferritin* H.C) in aorta tissue lysates of WT and PAR-1 KO mice were analyzed by RT-qPCR. Relative expression levels were normalized versus GAPDH. Results are representative of three independent experiments that yielded similar results. ^#^P < 0.05; ^##^P < 0.01 compared with WT mice.

The normal arterial endothelium regulates vascular tone through release of vasodilator, nitric oxide (NO). NO is stimulated with agents such as acetylcholine, ATP, bradykinin, and the calcium ionophore. Endothelial dysfunction, which is commonly defined as reduced endothelium-dependent vascular relaxation, occurs as an early event in atherosclerosis and hypertension^46,47^, and it has been reported PAR-1 is associated with endothelial-dependent vasomotor activity in various blood vessels^29,30^. To examine the role of PAR-1 in vasomotor modulation, we investigated acetylcholine-induced vasorelaxation in the isolated aortic rings of PAR-1^−/−^ mice compared with the littermate control. In aortic rings pre-contracted with U46619 (a synthetic agonist of thromboxane receptor), acetylcholine-induced vasorelaxation was lower in the aortic rings of PAR-1^−/−^ mice than in the littermate control (Fig. 9A). However, there was no significant difference in relaxation induced by SNP, a NO donor, in PAR-1^−/−^ mice compared with the littermate control (Fig. 9B).

Blood flow-induced vasodilation is known to contribute to vascular tone and basal blood pressure. We measured arterial blood pressure by tail-cuff method. The basal blood pressure was relatively high in PAR-1 KO compared to WT mice, but no significant differences in systolic and diastolic pressure were observed between PAR-1 KO and littermate control (Fig. 9C).

Next we focused on the mRNA inductions of eNOS, NQO1 and ferritin heavy chain (*Ferritin* H.C) (Fig. 9D). PAR-1 deficiency markedly inhibited mRNA expressions of KLF2- and Nrf2-targeted genes. Taken together, these results suggest PAR-1 regulates endothelial dysfunction *in vivo*.

### PAR-1 deficiency increases inflammatory response in atheroprotective region of aorta

**Figure 10:**
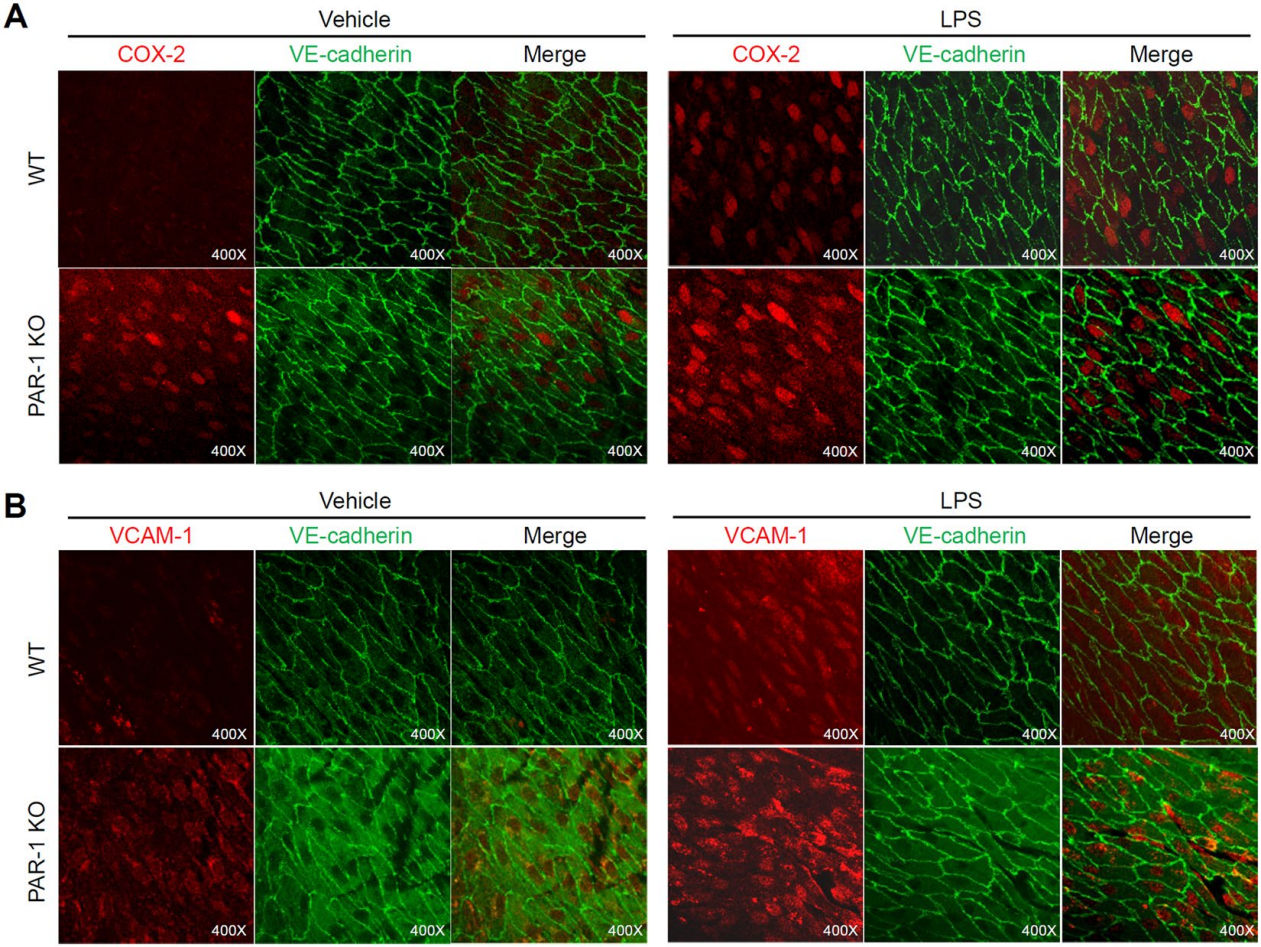
PAR-1 deficiency enhances inflammatory response in atheroprotective region. The expression of COX-2 (**A**) and VCAM-1 (**B**) in mouse aortic endothelium was analyzed by *en face* immunofluorescence staining assay using anti-COX-2 and anti-VCAM-1 antibody (red). Mice were treated with either 1 mg/kg of LPS or vehicle control by intraperitoneal injection. Endothelium of thoracic aorta was stained with anti-VE-cadherin antibody for endothelial cell-cell junction stating (green) and Topro3 for nuclear staining (blue), and photographed under a confocal microscope (400×).

Because we found that PAR-1 contributes to laminar flow-induced anti-inflammatory response *in vitro* (Fig. 7), we further investigated the role of PAR-1 on LPS-induced inflammation *in vivo* by using *en face* technique. It has been well established that thoracic region of aorta has steady laminar flow and is resistant to develop atherosclerosis compared to lesser coverture or bifurcation region^2,3^. Endothelial inflammatory response was evaluated by coimmunostaining with inflammatory markers and endothelial marker. As shown in Fig. 10, staining intensity of anti-cyclooxygenase-2 (COX-2) and anti-VCAM-1 was relatively high in atheroprotective region of aorta from PAR-1 KO compared to WT mice in resting state. In addition, lipopolysaccharide (LPS)-induced endothelial inflammatory response in thoracic region of PAR-1 KO was greater than that of WT mice. These results suggest that PAR-1 is involved in laminar flow-mediated atheroprotective response *in vivo*.

## Discussion

In this study, we sought to determine whether PAR-1 acts as a mechano-sensor in the laminar flow-mediated atheroprotective signaling pathway. It was found PAR-1 participates in the mediation of laminar flow-dependent cytoprotective effects in ECs. Our findings show that laminar flow stimulates PAR-1 which is internalized into early endosomes, and then PAR-1 activation triggers laminar flow-dependent atheroprotective gene expression by regulating Src, AMPK, ERK5, HDAC5, and eNOS. In addition, VCAM-1 expression and monocyte adhesion induced by TNFα were reduced by laminar flow, and these effects were prevented by the siRNA knockdown of PAR-1. Moreover, PAR-1 regulated laminar flow-mediated anti-apoptotic response. Finally, acetylcholine-induced vasorelaxation was markedly less for the aortic rings of PAR-1 knockout mice than for the littermate controls. These findings show PAR-1 is a key regulator of laminar flow sensing and mediates anti-atherosclerotic responses in ECs.

In this study, we found that PAR-1 expression was slightly downregulated by laminar flow although the majority of PAR-1 remains 24 hour later. During ligand-mediated plasma membrane receptor signaling, receptor can be recycled by the process of receptor internalization and small part of them undergo to lysosomal degradation pathway^48,49^. Thus, laminar flow-mediated PAR-1 downregulation might be physiological process of receptor activation rather than inhibitory process of laminar flow. However, it is not clear whether laminar flow-mediated PAR-1 activation is dependent on classical ligand. Previous reports have shown that endothelial protein C receptor (EPCR) interacts with APC and mediates APC-induced cytoprotective signaling through PAR-1 activation^27,50,51^. In particular, the occupancy of EPCR promotes PAR-1-dependent protective signaling of thrombin and APC^42^, and it has also been reported that thrombin-thrombomodulin complex, EPCR, and PAR-1 are localized in the lipid rafts of endothelial cells^42^. Our data indicate that PAR-1 was activated in response to laminar flow and internalized into early endosomes (Fig. 1). However, it is not known whether PAR-1 is able to interact with EPCR in the lipid rafts of endothelial cells or whether EPCR supports the cleavage of PAR-1 by laminar flow. Actually, we found that laminar flow-mediated endothelial signaling was inhibited by EPCR depletion as well (data not shown). These results suggest that PAR-1 might cooperate with EPCR for laminar flow-mediated mechanotransduction. Further studies will be required to analyze interaction between PAR-1 and EPCR in response to laminar flow.

HDAC5 is a class IIa histone deacetylase and negatively regulates the transcriptional activity of MEF2, which is a key player in laminar flow-induced KLF2 activation^21^. It has also been reported that laminar flow-induced phosphorylation and nuclear export of HDAC5 are regulated by a Ca^2+^/calmodulin-dependent pathway^20^, and that laminar flow enhances the expressions of KLF2 and eNOS by removing the inhibitory effect of HDAC5. In the present study, we found laminar flow-induced HDAC5 phosphorylation was inhibited by siRNA knockdown of PAR-1 (Fig. 5). However, the possible involvements of PAR-1 and HDAC5 signal transduction have not been established. Nevertheless, it has been shown that PAR-1 couples to heterotrimeric G proteins and stimulates MAP kinases and PKC, increases intracellular Ca^2+^ levels and promotes diverse cellular responses^52^, and that laminar flow-induced HDAC5 phosphorylation is regulated by Ca^2+^/calmodulin-dependent signaling^20^. Additional studies to determine CaM kinases would improve understanding of the PAR-1 protective mechanism.

In the present study, we used global PAR-1 knockout. In previous reports, researchers have created several PAR-1 KO strains to explore the role of PAR-1 in various pathological conditions/models. Connolly *et al*. found that about half of PAR-1^−/−^ mouse embryos died at mid-gestation due to multiple bleeding, and that survivors became grossly normal adult mice with no bleeding diathesis^53^. Several reports on PAR-1 KO showed that ischemia/reperfusion injury and vascular injury responses were attenuated in PAR-1 KO, which suggests PAR-1 plays the multiple roles during pathogenic conditions^54,55^. In the present study, PAR-1 deficiency was associated with reduced vasorelaxation by acetylcholine in the precontracted aorta (Fig. 8), which is similar to a previous report of endothelial NO-dependent relaxation by a PAR-1 agonist.

Laminar shear stress is known to induce the integrin-dependent signaling and ASF formation as early events of endothelial mechanotransduction. We found that PAR-1 regulated laminar flow-induced F-actin polymerization and phosphorylation of FAK and Src, suggesting the involvement of PAR-1 in early events of mechanotransduction. In addition, we also found that PAR-1 is important in laminar flow-mediated anti-inflammatory responses. The anti-inflammatory effect of laminar flow is regulated by the induction of transcription factors including KLF2, KLF4, and Nrf2^18,56,57^. In the current study, we found that KLF2 and Nrf2 induction of laminar flow were inhibited by PAR-1 depletion. These results indicated that gene regulation including KLF2 and Nrf2 is involved in PAR-1-mediated laminar flow signaling to anti-inflammatory response. Thus PAR-1 might modulate not only early event but also late response such as laminar flow-mediated anti-inflammatory responses through gene expression.

Summarizing, the present study identifies PAR-1 as a novel mechano-sensor of laminar flow. PAR-1 activation was found to be induced by laminar flow and to stimulate the expressions of atheroprotective genes via signaling pathways of Src, AMPK, ERK5, HDAC5, and eNOS. In addition, laminar flow-activated PAR-1 was observed to promote anti-inflammatory and anti-apoptotic responses, and acetylcholine-induced vasorelaxation was suppressed in the aortas of PAR-1^−/−^ mice versus the littermate controls. Based on these findings, we therefore propose PAR-1 be considered a novel pharmacologic target for the treatment of vascular diseases.

## Materials and Methods

### Cell culture and laminar flow

Human umbilical vein endothelial cells (HUVECs) were cultured in M200 medium (Gibco) containing 5% fetal bovine serum (FBS) and endothelial growth factor supplement (LSGS; Cascade biologics, Portland, OR, USA) on 0.2% gelatin coated cell culture dishes. Cells were used for experiments at passage 4, 5 and 6. U937 monocytes were grown in RPMI 1640 medium containing 10% FBS. Confluent HUVECs cultured in 100 mm dishes were exposed to laminar unidirectional flow (12 dynes/cm^2^) for 24 hours using a cone flow system in a 5% CO^2^ humidified incubator at 37 °C, as described previously^58^. Morphological changes were observed under a microscope at an original magnification of x100.

### Reagents and antibodies

Recombinant human TNF-α was purchased from R&D Systems (Wiesbaden-Norderstedt, Germany). Anti-PAR-1 (ATAP2), anti-NQO1, anti-eNOS, and anti-VCAM-1 were from Santa Cruz Biotechnology (Santa Cruz, CA, USA), and anti-phospho-HDAC5, anti-HDAC5, anti-phospho-ERK5, anti-ERK5, anti-phospho-AKT, anti-AKT, anti-phospho-AMPK, anti-AMPK, anti-phospho-eNOS, anti-EEA1, anti-phospho-Src, anti-phospho-FAK, anti-phospho-Erk1/2, anti-Erk1/2 and anti-VE-cadherin were from Cell Signaling Technology (Danvers, MA, USA). Anti-COX-2 was from Cayman Chemical Company (Ann Arvor, MI, USA). Anti-tubulin was from Sigma–Aldrich (St. Louis, MO, USA). Phenylephrine, Acetylcholine and U46619 were purchased from Sigma–Aldrich (St. Louis, MO, USA). Pertussis toxin (PTX) was purchased from Gibco. Small interfering RNA against human PAR-1 was purchased from Santa Cruz Biotechnology (sc-36663, Santa Cruz, CA, USA). For PAR-1 silencing, HUVECs were transiently transfected with 100 pmol/L of control RNA or siRNA targeting human PAR-1 using Lipofectamine 2000 reagent (#11668-019, Invitrogen, Carlsbad, CA, USA) according to the manufacturer’s instructions. A non-specific control siRNA from Bioneer was used as a negative control. HUVECs were harvested 48–72 hours after siRNA transfection, protein expressions were assessed by immunoblotting with antibodies and mRNA levels by quantitative real time RT-PCR (RT-qPCR).

### Monocyte adhesion

HUVECs were transfected with 100 pmol/L of control RNA or siRNA targeting human PAR-1. Three days after transfection, cells were treated with 10 ng/ml tumor necrosis factor-α (TNF-α) and then exposed to laminar flow (12 dynes/cm^2^) for 24 hours. Separately, U937 cells were treated with 5 μM Vybrant DiD and incubated for 20 minutes at 37 °C (V-22887, Invitrogen, Carlsbad, CA, USA), as described previously^59^. These cells were then washed twice, resuspended in serum-free medium (RPMI), added to HUVECs that had been cultured in 100 mm dishes and incubated for 1 hour at 37 °C (1 × 10^7^/100 mm dish). After removing media, non-adherent cells were removed by washing twice with phosphate buffered saline (PBS). Adherent cells were observed under a confocal fluorescence microscope (Leica, Bannockburn, IL, USA).

### TUNEL assay

Three days after transfecting HUVECs with siRNA, cells were cultured in serum-free medium and then stimulated with laminar flow (12 dynes/cm^2^) for 24 hours. Cells were then washed with PBS twice and fixed with 4% paraformaldehyde for 30 minutes. Apoptotic cells were detected using a TUNEL assay kit (Roche Diagnostics Australia, NSW, Austria). Nuclei were stained with DAPI, and TUNEL positive cells were observed by immunofluorescence.

### Western blotting

HUVECs were lysed with radioimmunoprecipitation assay lysis buffer supplemented with 1 mmol/L phenylmethylsulfonyl fluoride and 0.01 mmol/L PIC (protease inhibitor cocktail). Cell lysates were incubated on ice for 15 minutes and then centrifuged at 15,000 g for 10 minutes at 4 °C. Protein concentrations in samples were determined using supernatants and a Bradford assay. Proteins were separated by SDS-PAGE and transferred to poly-vinylidene difluoride membranes, which were immunoblotted with primary antibodies and then with corresponding secondary antibodies. Signals were visualized using electrochemiluminescence detection regents (EMD Millipore, Billerica, MA, USA), according to the manufacturer’s instructions.

### PAR-1 immunofluorescent staining

To visualize PAR-1 internalization by HUVECs, cells were exposed to laminar flow (12 dynes/cm^2^) for different times. Briefly, after washing with PBS, cells were fixed with 4% paraformaldehyde for 30 minutes, permeabilized for 5 minutes at room temperature, blocked using 5% goat serum in PBST, and incubated with anti-EEA-1, anti-PAR-1 (ATAP2), and anti-VE-cadherin antibodies for 18 hours at 4 °C. After washing twice with PBS, HUVECs were incubated with anti-mouse IgG conjugated with FITC and anti-rabbit IgG conjugated with TRITC (Invitrogen, Carlsbad, CA, USA, 1:1,000) for 90 minutes. Signals were observed using an immunofluorescence microscope.

### Quantitative real time RT-PCR

RT-qPCR was used to assess the mRNA expressions of the Nrf2 or KLF2 genes, as previously described^60^. Briefly, total RNA was isolated using TRIzol Reagent (Invitrogen, Carlsbad, CA, USA) and reverse transcription reaction was conducted by using TaqMan reverse transcription reagents (Applied Biosystems, Carlsbad, CA, USA) according to the manufacturer’s instructions. RT-qPCR was conducted with 1 μl of template cDNA and Power SYBR Green (Applied biosystems, Carlsbad, CA, USA) using an ABI PRISM 7500 (Applied Biosystems). Quantification was carried out using the efficiency-corrected ΔΔCq method. The primers used to amplify DNA sequences were as follows: NQO1 forward 5′-TTACTATGGGATGGGGTCCA-3′ and reverse 5′-TGCCAAAACTGTTCACCAAA-3′. Ferritin heavy chain forward 5′-TGACAAAAATGACCCCCATT-3′ and reverse 5′-GATGGCTTTCACCTGCTCAT-3′, KLF2 forward 5′-CCTCCCAAACTGTGACTGGT-3′ and reverse 5′-GAGGGAGAC CCTCTGTAGCC-3′, eNOS forward 5′-CCTGGAAAGTTCCCTCATCA-3′ and reverse 5′-CTTCTGGCAGGGAGACAGAC-3′, PAR-1 forward 5′-CTGTGGTGTATCCCATGCAG-3′ and reverse 5′-GCCAGACAAGTGAAGGAAGC-3′, GAPDH forward 5′-GGAGCCAAAAGGGTCATCAT-3′ and reverse 5′-GTGATGGCATGGACTGTGGT-3′.

### Flow cytometric analysis

Flow cytometry was used to analyze PAR-1 surface expression. After exposure to flow (12 dynes/cm^2^) for various times. HUVECs were harvested with 0.2% EDTA in PBS, fixed with 4% paraformaldehyde for 30 minutes at room temperature, blocked with 5% goat serum for 1 hour at room temperature and incubated with antibodies against PAR-1 (ATAP2) or anti-mouse IgG (a negative control) for 18 hours at 4 °C. After washing twice with PBS, cells were incubated for 1 hour at room temperature with anti-mouse IgG conjugated with TRITC, and analyzed by flow cytometry (BD Biosciences, Franklin Lakes, NJ, USA).

### Vascular reactivity study (tension response of aortic rings)

PAR-1 knockout mice (B6.129S4-F2rtm1Ajc/J) were purchased from Jackson laboratory (Stock Number: 002862). Thoracic aortas were removed from C57BL/6 mice and PAR-1 KO mice (12 weeks old) after sacrifice. Adventitial fat and connective tissues were carefully removed and arteries cut into 2-mm rings under a microscope. These rings were then suspended by a small vessel wire myograph containing 37 °C Krebs-bicarbonate buffer (117 mM NaCl, 4.8 mM KCl, 1.2 mM MgSO_4_, 25 mM NaHCO_3_, 1.2 mM KH_2_PO_4_, 5.7 mM glucose, 2.5 mM CaCl2) in a 95% O_2_/5% CO_2_ atmosphere. Isometric tension was measured using a force transducer. Aortic rings were equilibrated in buffer for 30 minutes and then constricted by adding U46619, a thromboxane receptor agonist, until a steady-state was reached. Endothelium-dependent and -independent relaxations were assessed by measuring the dilatory response of arteries to acetylcholine (from 10^−9^M to 10^−6^M) or sodium nitroprusside (from 10^−9^M to 10^−6^M), respectively. All animal experiments were conducted in accordance a protocol approved beforehand by the Institutional Animal Care and Use Committee of Yeungnam University College of Medicine, Daegu, Republic of Korea. In addition to this, all experiments were performed in accordance with the relevant guidelines and regulations.

### *en face* staining

To determine the role of PAR-1 in anti-inflammatory response *in vivo*, 12-week-old male C57BL/6 mice and PAR-1 KO mice were intraperitoneally treated with LPS (1 mg/kg of body weight) or vehicle control. Following euthanization, vascular perfusion was performed with saline for 5 min followed by fixation with 4% paraformaldehyde for 5 min. Isolated thoracic aorta was incubated with 0.1% PBS with Tween, and then fat was removed. 5% goat serum was used for blocking and antibody diluents. Aortic endothelial cells were stained with anti-VE-cadherin antibody and Topro3 for endothelial cell junction and nuclear, respectively. The expression of inflammatory markers was determined by immunofluorescence staining with anti-COX-2 and anti-VCAM-1 antibody under the Confocal microscope.

### Blood pressure measurements

Basal blood pressure (BP) was performed in conscious mice with an automated multichannel system by using tail cuff method followed by the manufactural instructions (CODA system; Kent Scientific Corporation). Mice were caged with blinded identity in a quiet environment and repeated measurements of basal systolic arterial pressure (SAP) and diastolic arterial pressure (DAP). Data was digitally recorded on a computer.

### Statistical analysis

Results are presented as the means ± SDs of three independent experiments. The statistical analysis was performed using the Student’s *t-*test, and probability values (*P* values) of < 0.05 were considered statistically significant.

## Data Availability

All data generated or analysed during this study are included in this published article.
